# Rivastigmine modifies the α-secretase pathway and potentially early Alzheimer’s disease

**DOI:** 10.1038/s41398-020-0709-x

**Published:** 2020-02-03

**Authors:** Balmiki Ray, Bryan Maloney, Kumar Sambamurti, Hanuma K. Karnati, Peter T. Nelson, Nigel H. Greig, Debomoy K. Lahiri

**Affiliations:** 1 Department of Psychiatry, Laboratory of Molecular Neurogenetics, Indiana University School of Medicine, Indianapolis, IN 46202 USA; 2 Indiana Alzheimer Disease Center, Indiana University School of Medicine, Indianapolis, IN 46202 USA; 3 Department of Neurosciences, Medical University of South Carolina, Charleston, 29425 SC USA; 4 National Institute on Aging, Drug Design and Development Section, Bethesda, MD 20892 USA; 5 Sanders-Brown Center on Aging, University of Kentucky, Lexington, KY 40536 USA; 6 Department of Medical and Molecular Genetics, Indiana University School of Medicine, Indianapolis, IN 46202 USA

**Keywords:** Health sciences, Diseases, Psychiatric disorders

## Abstract

Rivastigmine (or Exelon) is a cholinesterase inhibitor, currently used as a symptomatic treatment for mild-to-moderate Alzheimer’s disease (AD). Amyloid-β peptide (Aβ) generated from its precursor protein (APP) by β-secretase (or BACE1) and γ-secretase endoproteolysis. Alternative APP cleavage by α-secretase (a family of membrane-bound metalloproteases– Adamalysins) precludes the generation of toxic Aβ and yields a neuroprotective and neurotrophic secreted sAPPα fragment. Several signal transduction pathways, including protein kinase C and MAP kinase, stimulate α-secretase. We present data to suggest that rivastigmine, in addition to anticholinesterase activity, directs APP processing away from BACE1 and towards α-secretases. We treated rat neuronal PC12 cells and primary human brain (PHB) cultures with rivastigmine and the α-secretase inhibitor TAPI and assayed for levels of APP processing products and α-secretases. We subsequently treated 3×Tg (transgenic) mice with rivastigmine and harvested hippocampi to assay for levels of APP processing products. We also assayed postmortem human control, AD, and AD brains from subjects treated with rivastigmine for levels of APP metabolites. Rivastigmine dose-dependently promoted α-secretase activity by upregulating levels of ADAM-9, -10, and -17 α-secretases in PHB cultures. Co-treatment with TAPI eliminated rivastigmine-induced sAPPα elevation. Rivastigmine treatment elevated levels of sAPPα in 3×Tg mice. Consistent with these results, we also found elevated sAPPα in postmortem brain samples from AD patients treated with rivastigmine. Rivastigmine can modify the levels of several shedding proteins and directs APP processing toward the non-amyloidogenic pathway. This novel property of rivastigmine can be therapeutically exploited for disease-modifying intervention that goes beyond symptomatic treatment for AD.

## Introduction

Alzheimer’s disease (AD) is the most common cause of dementia and the fifth leading cause of death in the elderly^1^. Deposition of the amyloid-β (Aβ) peptide of 4.2 kDa within the brain parenchyma and hyperphosphorylation of the microtubule-associated protein, tau (MAPT) of 42–47 kDa, are cardinal neuropathologies of AD^2,3^. To generate Aβ, a type 1 integral membrane protein, APP, is sequentially cleaved by β-secretase 1 (BACE1) into two fragments: secreted APPβ (sAPPβ) and a membrane-bound cytoplasmic tail fragment β (CTFβ) of 99 amino acids, which is further processed by γ-secretase, a complex of four integral membrane subunits. Aβ secreted from cultured cells varies in length from 36 to 43 amino acids). Overall processing of APP to Aβ and subsequent risk of AD is a product of genetic and environmental influences that can coalesce through epigenetic processes^4,5^. Aβ is normally secreted into cell culture media, cerebrospinal fluid, plasma and the vitreous humor^6^, but it represents a minor fraction of peptides derived from γ-secretase cleavage of APP. Instead, the majority of APP is processed by α-secretase at a site within the Aβ peptide sequence to a longer secreted APPα (sAPPα) and CTFα (83 amino acids), which is further cleaved by γ secretase to create a shorter 3 kDa fragment, P3. Alterations in levels of Aβ, P3, and other APP processing products can accompany other causes of dementia, such as normal pressure hydrocephalus (NPH)^7^. Hence, redirection of APP processing towards the α-secretase pathway may be of potential therapeutic value in AD. Therefore, in this article, we demonstrate a non-cholinergic property for the anti-AD drug rivastigmine. Specifically, rivastigmine reduces Aβ generation by directing APP processing towards the neuroprotective pathway dominated by the α-secretase pathway.

The primary α-secretases are known as ADAMs (A Disintegrin And Metalloproteases), a family of integral membrane proteins with roles that drive the ectodomain shedding of key transmembrane proteins such as the Notch receptor, APP, TNF-α, ErbB2, and ErbB4^8^. Notably, ADAM-9, -10 and -17 are involved in the cleavage of APP as a redundant family of α-secretases. ADAM-17 is also involved in the shedding of pro-TNF-α to produce TNF-α, and can, therefore, regulate this important pro-inflammatory pathway^9^. ADAM-17 inhibitor (BMS 561392) treatment does not increase levels of Aβ peptides^10^. This is most likely due to distinct compartmentalization of APP by transport to the cell membrane (location of α-secretase processing) or endosomes (location of β-secretase processing)^11^.

Rivastigmine (aka, ENA 713/carbamoylatine), an FDA-approved acetyl- and butyryl-cholinesterase inhibitor (AChEI/BuChEI), is used to treat mild-moderate AD by elevating synaptic acetylcholine (ACh) levels, under the trademark “Exelon”. As of 2015, at least 13 double-blind clinical trials of rivastigmine efficacy for AD^12^ demonstrated the agent to be beneficial for mild-to-moderate AD, as compared to placebo. In addition, rivastigmine has been involved in 137 studies registered with the US National Library of Medicine as of the composition of this paper. These include not only symptomatic relief for AD, but also potential treatment of post-operative delirium, amnestic mild cognitive impairment (MCI), Down syndrome, cocaine and amphetamine dependence, and Parkinson’s disease, among others^13^. Although rivastigmine is a dual cholinesterase inhibitor (ChEI), it more efficiently binds BuChE than AChE^14^. It has been suggested that preserving levels of extracellular acetylcholine through inhibiting BuChE could play an important role in treating AD^15^. However, evidence has emerged that rivastigmine may not merely be symptom-ameliorating but may provide disease-modifying activity^16–18^.

**Fig. 1:**
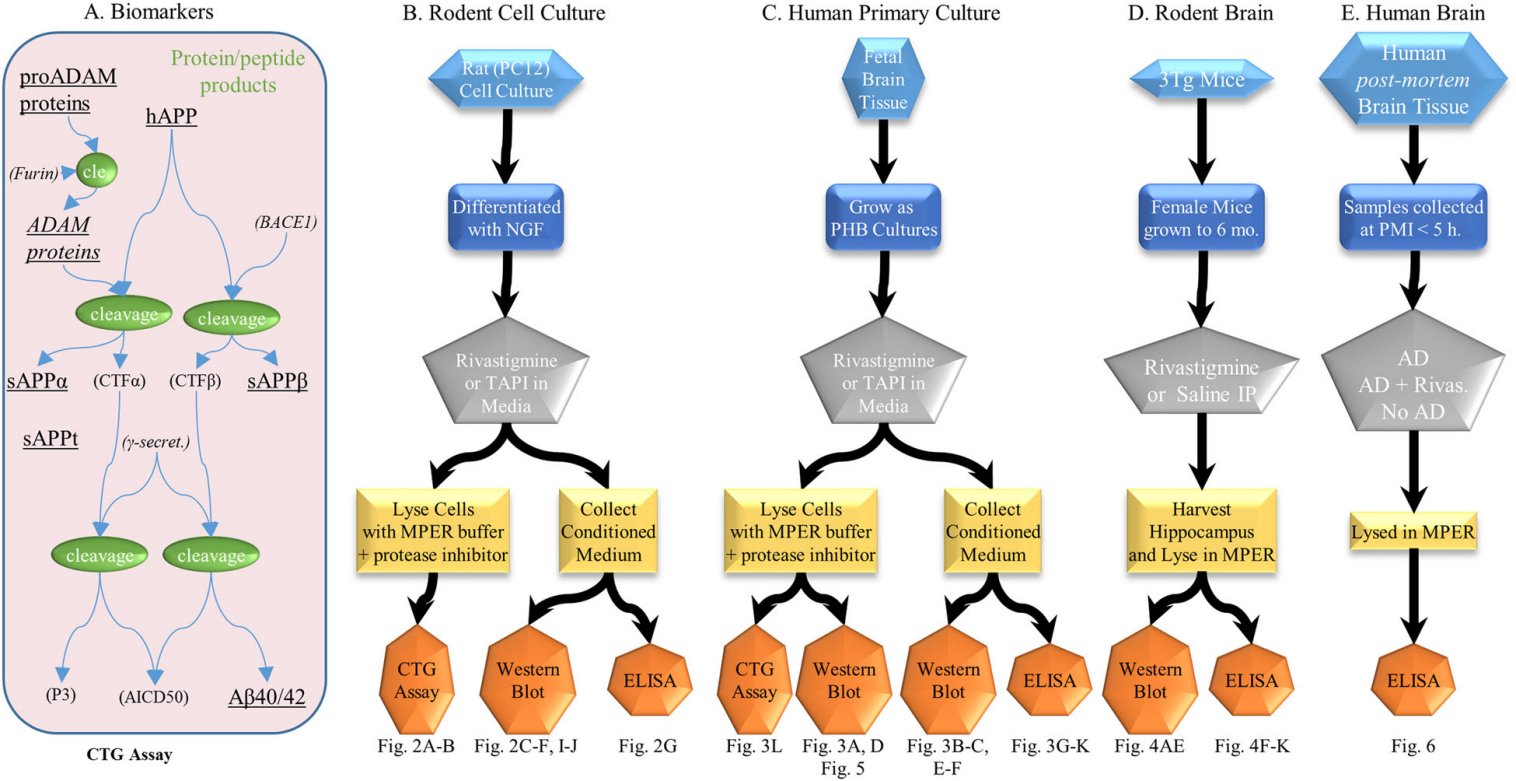
Overview of the study and Research workflow. This report used four complementary materials and workflows to elucidate rivastigmine activity. **a** Interrelations of APP pathway metabolites and biomarkers (some are studied herein). Briefly, hAPP serves as substrate for several enzymes. Among these are the *ADAM proteins*, which begin as their *proADAM protein* counterparts, which are cleaved by *Furin*. hAPP is also cleaved by *BACE1*. The cleavages of hAPP produce the protein/peptide products sAPPα, sAPPβ, CTFα, and CTFβ. The total of sAPPα and sAPPβ, along with minor cleavage products, produce sAPPt. Cleavage of CTFα and CTFβ by *γ-secretase* complex produces P3 from CTFα, Aβ40 and Aβ42 from CTFβ, and AICD50 from both CTFα and CTFβ. In addition, the CTG assay assesses overall cell culture health. **b** Rat pheochromocytoma (PC12) cell cultures were neuronally differentiated with NGF and then treated with Rivatigmine and TAPI, alone and in combination in media. Conditioned media (CM) was collected and cells were harvested and lysed. Lysate was used to assess cell culture health by CTG, while CM was assayed for protein and peptide levels by western blot (WB) and ELISA. **c** Primary human brain (PHB) cultures were grown according to protocols developed in our laboratory. PHB cultures were treated with rivastigmine and TAPI, alone and in combination. CM was collected and cells harvested and lysed. Culture health was assessed by CTG, and both lysates and CM were used in western blots and ELISA. **d** Female 3 × Tg mice were grown to 6 months and injected IP with rivastigmine or saline. Hippocampus was harvested and lysed to use in western blots and ELISA. **e** Donated human autopsy brain tissue was collected from patients who had AD without drug treatment, AD with rivastigmine treatment, and non-AD subjects. Tissues were lysed and levels of peptides and proteins assayed by ELISA.

While rivastigmine is approved as a ChEI, it may be that its overall efficacy involves acting on other pathways. We have previously demonstrated that select ChEIs possess APP-modulating properties^19^, but such action appears to be via mechanisms distinct from their cholinergic activities. For example, the ChEI (−)-Phenserine and its cholinergically inert opposite enantiomer (+)-Phenserine (posiphen) both have APP and Aβ lowering properties in cell culture, animal models^20^, and AD subjects^21^. In addition, donepezil elevates sAPPα and reduces Aβ in culture by upregulating sorting nexin protein 33 (SNX33)^22^. Rivastigmine also elevated sAPPα and lowers levels of Aβ in a rodent primary culture system, as well as enhancing levels of axonal markers such as synaptosomal-associated protein- 25 (SNAP25)^17^. Herein we employed a diverse but integrated approach to test our hypothesis, which included (i) rat neuronal PC12 cultures, (ii) primary human brain (PHB) cultures^23^, (iii) triple-transgenic AD model mice, and (iv) postmortem human brain samples from AD patients who had not been exposed to rivastigmine, patients treated with rivastigmine, and non-AD age-matched controls (Fig. 1).

Rivastigmine induced a dose-dependent shift toward α-secretase processing of APP in PHB cultures and transgenic animals. This shift was also reflected in postmortem human brain samples collected from subjects treated with rivastigmine-associated change. We characterized this as a shift in processing because levels of overall APP were not altered by rivastigmine. In addition, we measured effects of rivastigmine on the ADAM α-secretase proteins and observed increases in levels of proproteins and mature proteins. Our results demonstrate that rivastigmine can modify levels of the active form of several α-secretases and redirect APP processing to the non-amyloidogenic pathway. This supports the notion that rivastigmine potentially possesses disease-modifying activity and opens the door to investigate rivastigmine derivatives that have low ChEI activity to potentially support higher dosing without the accompanying undesired effects of modifying cholinesterase (ChE) activity. Rivastigmine’s non-cholinergic effects on AD have not been reported much in literature specific to AD patient outcomes. We interpret this to mean that rivastigmine’s non-cholinergic effects would not be expected to be evident at stages where the drug would be prescribed. Rivastigmine is prescribed to treat mild-to-moderate dementia. The consensus of the field is that such stages may be too late to apply disease-modifying treatments^24–26^. We contend, in agreement with this trend, that any disease-modifying outcomes associated with rivastigmine have been effectively hidden by the disease stages in which the drug is typically prescribed.

In the present work, we demonstrate rivastigmine’s novel property of directing APP processing into the non-amyloidogenic pathway in a comprehensive manner, utilizing relevant cell cultures, transgenic animal model, and human samples from extreme ends of the lifespan (Fig. 1).

## Materials and methods

### Rivastigmine

Rivastigmine was provided as a gift by Dr. Martin Farlow (Indiana University School of Medicine) as 1.5 mg (rivastigmine tartrate) pharmaceutical capsules. Capsule contents were suspended in sterile water and disrupted by sonication. Suspensions were clarified by centrifugation to yield 5 mM stock solution. Other capsule contents included hydroxypropyl methylcellulose, magnesium stearate, microcrystalline cellulose, and silicon dioxide, generally considered pharmaceutically inert^27^. Concentration of stock solution was verified by comparison to analytical standards (Novartis, East Hanover, NJ) by UV-Vis spectrometry with Nanodrop spectrophotometer (ThermoFisher Scientific, Waltham, MA)

### Neuronally differentiated pheochromocytoma (PC12) cell culture

PC12 cells were obtained from ATCC, and cultured as described in our previous work^28^. Cells were differentiated by exposure to nerve growth factor (NGF) (30 ng/ml) (Sigma) for 3 days in low (1%) serum containing RPMI1640 medium (ThermoFisher). Cell cultures were routinely assessed for mycoplasma contamination by use of a commercially available assay. STR profiling was not routinely performed over the course of these experiments. For cell culture experiments, sample size was determined by multiple previous works in cell culture reasonable sample sizes for our APP and other assays^29–32^.

### Drug treatments of differentiated PC12

On culture day 12, media were removed from differentiated PC12 cells, and different doses of rivastigmine (5, 10, and 20 µM) were mixed with fresh medium and added to the wells. Four additional wells were treated with 10 μM TAPI. Four further wells were co-treated with 10 or 20 μM of rivastigmine and 10 μM TAPI. After 4 days of drug treatment, conditioned media (CM) samples were collected and stored at −80 °C. Cells were then washed once with ice cold PBS, lysed by addition of mammalian protein extraction reagent (M-PER buffer, Pierce), and supplemented with protease inhibitor cocktail (Roche). Cell lysate (CL) samples were used for Cell Titer Glow (CTG, Promega) assays.

### Primary PHB culture

PHB cultures were prepared from the brain tissue of aborted fetuses (80–100 days gestational age). Tissue was obtained from the Birth Defects Research Laboratory (BDRL) of the University of Washington, Seattle, WA. BDRL is in compliance with all relevant State and Federal regulations. IRB approvals to handle and process such materials were obtained from Indiana University School of Medicine (IUSM), Indianapolis. Materials (10–20 g) were shipped to IUSM overnight in chilled Hibernate-E medium (Invitrogen), supplemented with B27 cell culture supplement, GlutaMax, and an antibiotics mixture. Cell culture procedures followed our previously described protocols^23^. Briefly, tissue was placed in a trypsin EDTA tube and kept on a shaking water bath (150 RPM) at 37 °C for 15 min. The trypsin-digested tissue was transferred to Hibernate-E medium in a 15 mL PET and triturated using a siliconized, fire-polished pipette several times followed by centrifugation at 400 × *g* for 15 min. The cell pellet was resuspended in Hibernate-E medium followed by one more trituration and centrifugation. The resulting pellet was resuspended in culture medium and cell counting was performed by the Trypan blue exclusion method. The cells were plated on poly-D-lysine coated 24-well plates in neurobasal medium, supplemented with 1× B27 + 0.5 mM glutamax + 5 ng/mL bFGF + antibiotics cocktail (neuro+). Half of the medium was replaced by fresh medium + supplements on every 4th day. We have previously established that PHB cultures grown under these conditions to produce a mixture of neuronal and astrocytic cells on culture day 20^31^. We, thus, consider them a sufficiently good working model for human brain cell studies. For cell culture experiments, sample size was determined by multiple previous works in cell culture reasonable sample sizes for our APP and other assays^29–31^.

### Drug treatments of PHB cultures

On the 20th culture day, medium was pipetted off PHB cultures and different doses of rivastigmine (100 nM, 1 µM, and 10 µM) were mixed with fresh neurobasal medium and added to wells. Four additional wells were treated with 10 μM TAPI. Four further wells were co-treated with 10 μM rivastigmine and 10 μM TAPI. Drug treatments were undertaken over 4 days and (Supplementary Fig. S1), after treatment, CM samples were collected and stored at −80 °C. Cells were then washed once with ice cold PBS, lysed by addition of M-PER buffer (Pierce), and supplemented with protease inhibitor cocktail (Roche). CL samples were used for the subsequent Western immunoblotting and other assays (Fig. 1).

### Cell viability assays

Equal volumes of CL samples were pipetted into white translucent 96-well plates (Corning) and an equal amount of CTG reagent was added (Promega). Plates were immediately placed on a shaker for 1 min followed by incubation at room temperature for 10 min. Thereafter, luminescence signals were recorded using a luminometer, as described previously^33^.

### Western blotting

CM protein samples were mixed with 2 × Laemmli buffer, boiled for 5 min and equal volumes (12 μL) were loaded onto a 26-well Criterion gel (BioRad) and electrophoresed for 1.2 h at 200 volts, then transferred onto a PVDF membrane (BioRad). After blocking with 5% milk in TBST, we probed with mAb6E10 antibody (BioLegend, San Diego, CA) to evaluate sAPPα levels. After several washes with TBST, the membrane was exposed 1 h to anti-mouse secondary IgG followed by further washes with TBST. Electrochemiluminescence (ECL) substrate (Pierce) was added, followed by exposure to X-ray film band densities. Thereafter, western immunoblot “Reprobe buffer” (Thermo) was added to the same strip of the membrane. Following vigorous shaking for 10 min, washes with TBST and blocking for 30 min with 5% milk, the strip was probed for 45 min with anti-mouse secondary IgG and then exposed to X-ray film to ensure that the sAPPα primary antibody had been completely removed. On confirming this, the strip was used to determine total sAPP band densities using mAb22C11 antibody (Chemicon). Western immunoblotting of the CL samples was done with 22C11 (for intracellular full-length APP), ADAM-9 (Sigma), ADAM-10 (Sigma), and ADAM-17 (Enzo life science) antibodies. Films were scanned at 16 bits (grayscale), 300dpi, without tone or color correction. Densitometry was measured by ImageJ^34^. CL signals were normalized to β-actin signals from the same lane, while CM and intracellular signals were analyzed without further normalization.

### ELISA procedure

Sensitive ELISAs were used according to the manufacturer’s protocols. Equal volumes (25 μL) of CM samples were incubated in wells, pre-coated with anti-human sAPPα antibody (2B3, from IBL America, Minneapolis, MN). This kit used anti-human APP (R101A4) as a detection antibody. Levels of Aβ peptides (1–40 and 42) were quantified using commercially available kits (IBL). For sAPPβ ELISA, anti-human sAPPβ-wild-type rabbit IgG was applied as a capture and R101A4 utilized as a detection antibody.

### Treatment of APP/PS1/tau Tg mice with rivastigmine

All experiments that used animals were reviewed and approved before initiation by the Institutional Animal Care and Use Committee (IACUC) of the Intramural Research Program, National Institute on Aging (Protocol No. 331-TGB-2021), which monitored all processes for compliance with appropriate regulatory and ethical standards. 3×TgAD mice were generated from a presenilin-1 mutant (PS1M146V) mouse embryo that was transfected with two expression plasmids: A cDNA encoding the “Swedish” double mutation of APP (APPKM670/671NL) and a tau mutation (MAPTP301L) that causes frontotemporal lobe dementia, both under the control of the Thy-1 promoter^35^. The 3×TgAD mice used in the present study had been backcrossed for 8 generations onto a C57BL/6 background and developed Aβ and tau pathologies and behavioral deficits over a longer time, compared to the original 3×TgAD mice^35^. Female 3×TgAD mice (weight range 26–46 g, 6 months) were randomized into treatment and control groups by online utility (random.org) and administered rivastigmine (0.75 mg/kg fresh in physiological saline, 100 µl/10 gm body weight, via daily IP injection for 21 days) or saline, respectively. The method of euthanasia was anesthesia/suffocation by CO_2_ followed by mechanical decapitation after complete unconsciousness, as checked by tail pinch method, consistent with the recommendations of the American Veterinary Medical Association. Animals were euthanized 90 min following their final dose, and their brains rapidly removed and dissected on wet ice. Investigators were not blinded to group allocation. We chose a sample size of five animals per group and assumed an attrition rate of one animal, which provided us with a worst-case of four animals per group. This sample size had previously proved adequate to detect Cohen’s *d* of >1^20,36^.

### Preparation of mouse brain lysates

Hippocampi were harvested from the mice by dissection followed by snap freezing in liquid nitrogen and storage at −80 °C until analysis. Secreted proteins from samples were isolated as previously^37^ with minor modification. Briefly, samples were homogenized in 50 mM NaCl, 0.2% DEA using a Polytron homogenizer (Kinematica GmbH, Germany), followed by centrifugation at 100,000 × *g* for 1 h. Supernatant (secreted fraction) was stored for analyses. M-PER buffer was added to pellet, sonicated briefly, and centrifuged at 13,000 × *g* for 15 min, then used to analyze intracellular soluble proteins.

### Analyses of mouse brain lysates

Protein concentration from secreted and intracellular soluble fractions was estimated by Bradford Assay. Then equal protein amounts of samples were loaded onto a Bis-Tris minigel, and western immunoblotting was performed as described above. Levels of sAPPt and sAPPα in the secreted fraction of the lysate were evaluated by using mAb22C11 and mAb6E10 antibodies, respectively. Aβ in all mouse tissues was measured by ELISA (IBL America)

### Human postmortem brain tissue analyses

Human postmortem brain tissue samples were obtained from the University of Kentucky Alzheimer’s Disease Center biobank using methods described previously^38^, and with IRB approvals from the University of Kentucky, Lexington, KY, USA^38^. Informed consent or assent was collected and is held at the University of Kentucky. Potential subjects were excluded a priori if they had severe neuropsychiatric or substance abuse disorders. Subjects were excluded postmortem if the brain regions were in an infarct or neoplasia (primary or metastatic) in the brain^38^. Subjects were clinically evaluated on an annual basis, drug use was monitored by self-report, and participants were asked to bring their medicine containers with them to each visit. Brain tissue samples were collected soon after death (postmortem interval <5 h), and a subset of subjects who were regularly treated with rivastigmine was identified. Samples were anonymized before distribution by the biobank. Samples from individuals who did not have AD or other neurological disorders are referred to as “non- AD controls” in this paper. A subsample of AD patients who did not take any ChEI medications were also included in the study. Samples were all from Brodmann areas 21/22, also known as the middle and superior temporal gyri. Following protein extraction, all samples were subjected to ELISA to evaluate levels of sAPPα, sAPPt and Aβ peptides (both 1–40 and 42). The same methods were employed as described above for the mouse brain lysates to extract presumed secreted and intracellular fractions from human postmortem brain tissue samples (Fig. 1). Each brain specimen cohort had five specimens per group. This sample size is sufficient at power 85% to detect a Cohen’s d of >2, with type I error rate (alpha) of 5%.

### Statistical analyses

Results were analyzed with the R statistical language, using generalized linear models (GLM)^39^ followed by analysis of deviance F test (ANODE/ANOVA) and simultaneous pairwise comparisons of estimated marginal means^40,41^ if predictor variable was nominal. GLMs relax assumptions of the traditional ANOVA, particularly assumptions of “normal” distribution and homoscedasticity (equal variances). Dose response analyses were on log-transformed µM dose + 1 to permit log(dose = 0) to be 0, instead of “undefined”. Effect sizes and calculations are described in Supplemental Methods. Briefly, FC: Fold-change, ΔFC: change of fold-change between rivastigmine and rivastigmine + TAPI treatments.

## Results

### Rivastigmine improved cell viability, and altered APP metabolism in rodent-origin neuronally differentiated cell cultures

**Fig. 2:**
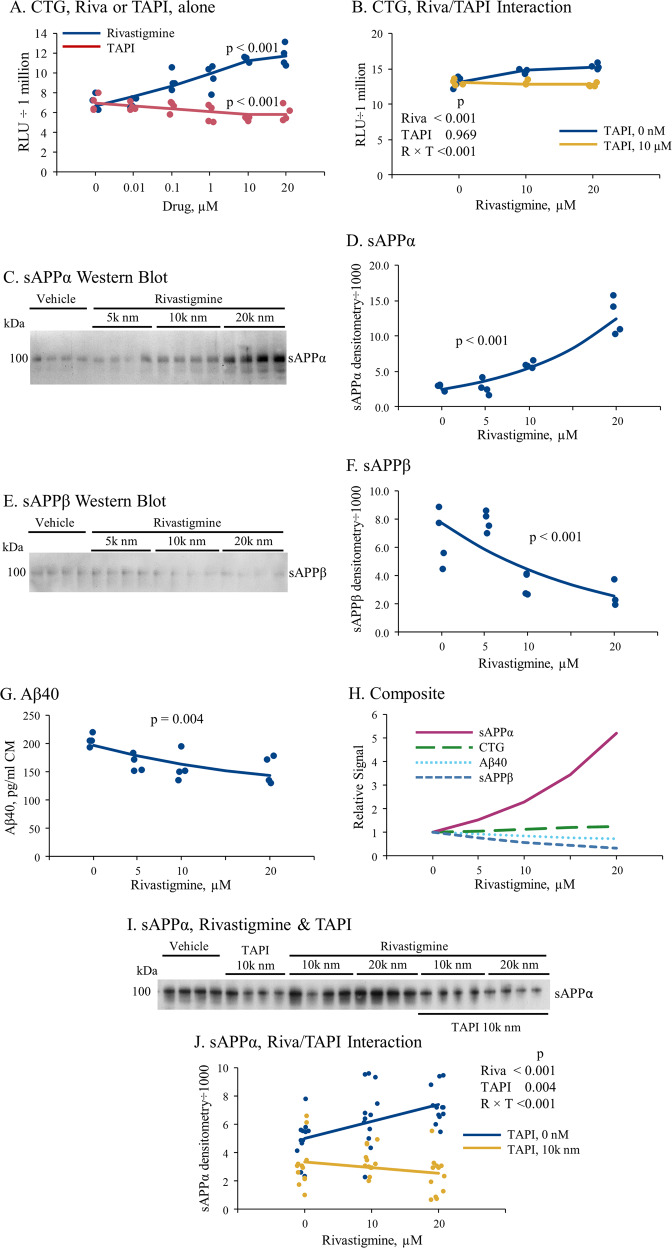
Effects of Rivastigmine and TAPI treatments on cell viability, Aβ40, and sAPPα in neuronally differentiated PC12 cells. Neuronally differentiated PC12 cultures were treated in two separate experiments with rivastigmine (A, C–H) or rivastigmine plus TAPI (B, I–J). **a** Culture viability assessed by CTG for Rivastigmine and TAPI. **b** CTG for Rivastigmine/TAPI co-treatment. **c** Western blot of sAPPα from PC12 cultures treated with three doses of rivastigmine plus vehicle. **d** Densitometry of blot was analyzed by GLM vs rivastigmine dose. **e** Western blot of sAPPβ from PC12 cultures treated with three doses of rivastigmine plus vehicle. **f** Densitometry of blot was analyzed by GLM vs rivastigmine dose. **g** Aβ40 from cells assayed by ELISA. **h** Composite plot of relative levels of each biomarker in A, D, F, and G. **i** Western blot of sAPPα from rivastigmine/TAPI co-treatment. **j** Densitometry of blot. Data from three blots were analyzed in J with mixed-level GLM, using random intercepts for each blot to account for inter-blot variation. A representative blot is shown. Aβ40 was measured by rodent-specific ELISA.

We observed a significant (*p* < 0.05) increase in rivastigmine-treated PC12 cell viability, as measured by CTG after rivastigmine treatment, whereas TAPI treatment reduced cell viability (Fig. 2a). When interaction of rivastigmine and TAPI was tested, TAPI eliminated the rivastigmine effect (Fig. 2b, *p* < 0.001 for interaction). We measured levels of secreted APP metabolites from the same samples. Rivastigmine treatment increased sAPPα (Fig. 2c, d) and reduced sAPPβ and Aβ40 levels (Fig. 2e–g) in CM samples. When we overlaid CTG, sAPPα, sAPPβ, and Aβ data (Fig. 2h), sAPPα elevation outstripped CTG gains, while Aβ40 and sAPPβ both were reduced in a dose-dependent fashion. We concluded that the apparent elevation of sAPPα was not likely to be solely due to overall increases in culture cell number. TAPI treatment (Fig. 2i, j) reduced sAPPα only-modestly in vehicle-treated controls, but fully reversed the rivastigmine-stimulated sAPPα to levels lower than untreated control cultures. ANOVAs are summarized in Supplementary Table S1. We had previously determined that rivastigmine treatment of primary rat brain cell cultures (PRBC) substantially preserved neuronal structure and protected neurons from degeneration while maintaining levels of neuronally-originated APP forms^17^.

### Rivastigmine treatment did not alter levels of intracellular full-length APP (holo-APP/hAPP) or total secreted APP (sAPPt) in PHB cultures but altered several individual APP processing product levels

**Fig. 3:**
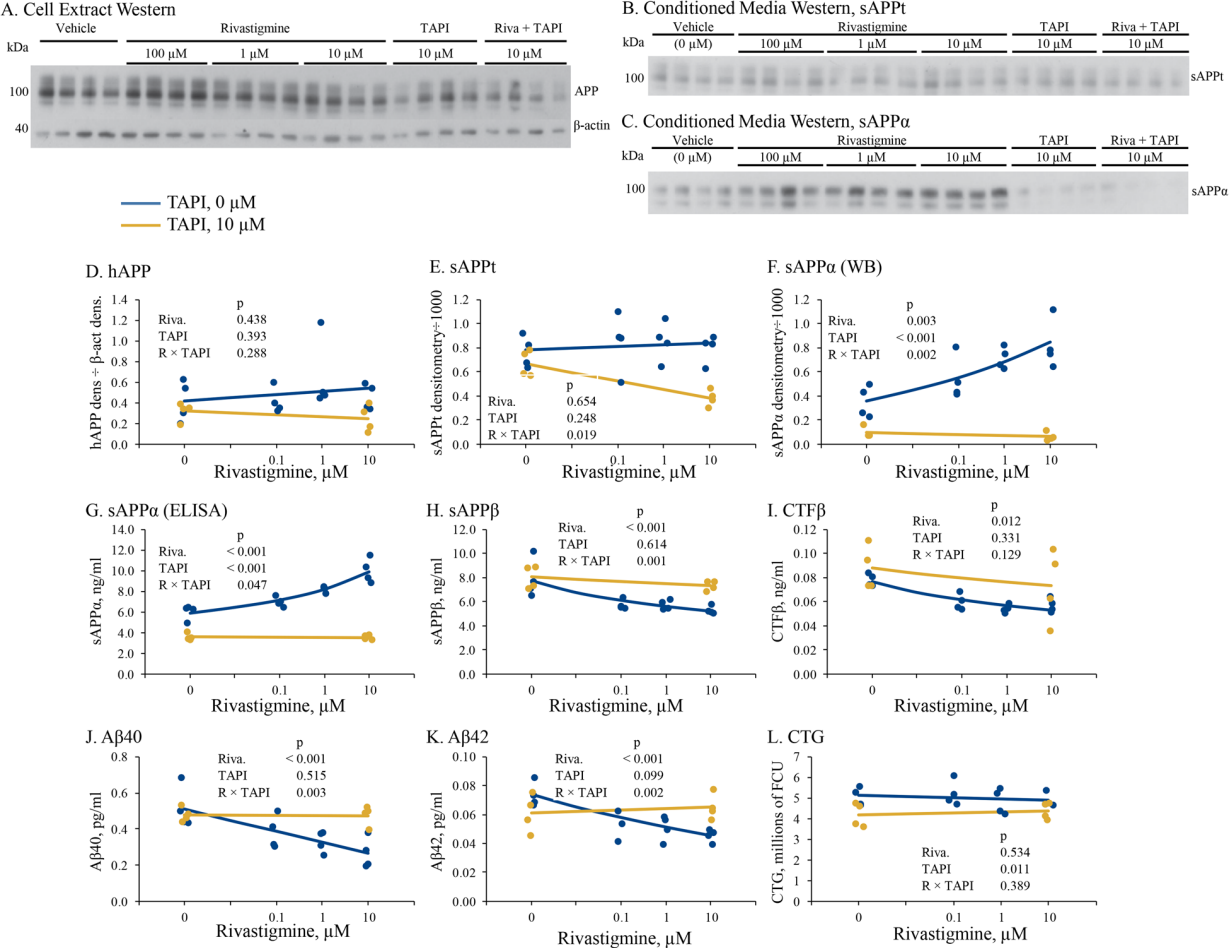
Effects of treatments on APP processing products and viability in human cultures, interaction effects. Primary human brain (PHB) cultures were co-treated with rivastigmine and TAPI as described in the text. Figure shows effects of rivastigmine or TAPI alone. **a** Cell extracts were prepared and probed for hAPP by western blot. **b** Conditioned media were analyzed on westerns probed separately for sAPPt, and **c** sAPPα. Blots were analyzed by semiquantitative densitometry. Figure shows combined effects of rivastigmine and TAPI. Densitometry analysis for **d** hAPP, **e** sAPPt, and **f** sAPPα. Different ELISAs were used to assay levels of **g** sAPPα (ELISA), **h** sAPPβ, **i** CTFβ, **j** Aβ40, and **k** Aβ42. **l** Cell viability was assessed with CTG.

We co-treated PHB cultures with Rivastigmine and TAPI and performed western blot analysis of hAPP from PHB lysates (Fig. 3a) and total secreted APP (sAPPt), and sAPPα (Fig. 3b, c). The latter blots were of conditioned media and, therefore, had no β-actin loading control. We performed independent ELISA for levels of sAPPα, sAPPβ, cytoplasmic fragment β (CTFβ), Aβ40, and Aβ42, as well as CTG assay. No significant effects appeared for rivastigmine or TAPI treatment on hAPP, nor for the interaction of rivastigmine and TAPI (Fig. 3d, Supplementary Table S2). Neither rivastigmine nor TAPI significantly altered total secreted APP (sAPPt) levels, but the interaction was significant (Fig. 3e, Supplementary Table S2). In essence, the very slight increase in sAPPt levels induced by rivastigmine (+0.006 fold-change) was more than reversed by TAPI treatment. By contrast, sAPPα (Fig. 3f, Supplementary Table S2) significantly increased (+0.138 FC/ + 0.039 FC) following rivastigmine treatment and significantly decreased (−0.074 FC/−0.039 FC) after TAPI treatment by both western blot and ELISA (Fig. 3g), and their interaction (ΔFC −0.175/−0.070) was also significant in that TAPI more than eliminated any rivastigmine effects. Separate ELISA for sAPPβ, APP cytoplasmic fragment (CTF)β, and Aβ40 and Aβ42 peptides (Fig. 3h–k, Supplementary Table S2) revealed that rivastigmine treatment significantly (*p* ≤ 0.001) reduced all four of these β-secretase processing products. TAPI did not induce a significant effect on sAPPβ, Aβ40, or Aβ42 in control cultures but it reversed the rivastigmine-mediated reduction of these APP-derived fragments. Cellular viability of PHB cultures (evaluated by CTG assay) was unaltered following rivastigmine treatment. TAPI caused a small (−0.018 FC) but significant (*p* = 0.011) decline in cellular viability (Fig. 3l), but rivastigmine appeared to prevent TAPI toxicity.

### Rivastigmine treatment elevated levels of sAPPt and sAPPα in hippocampus of 3×Tg mice

**Fig. 4:**
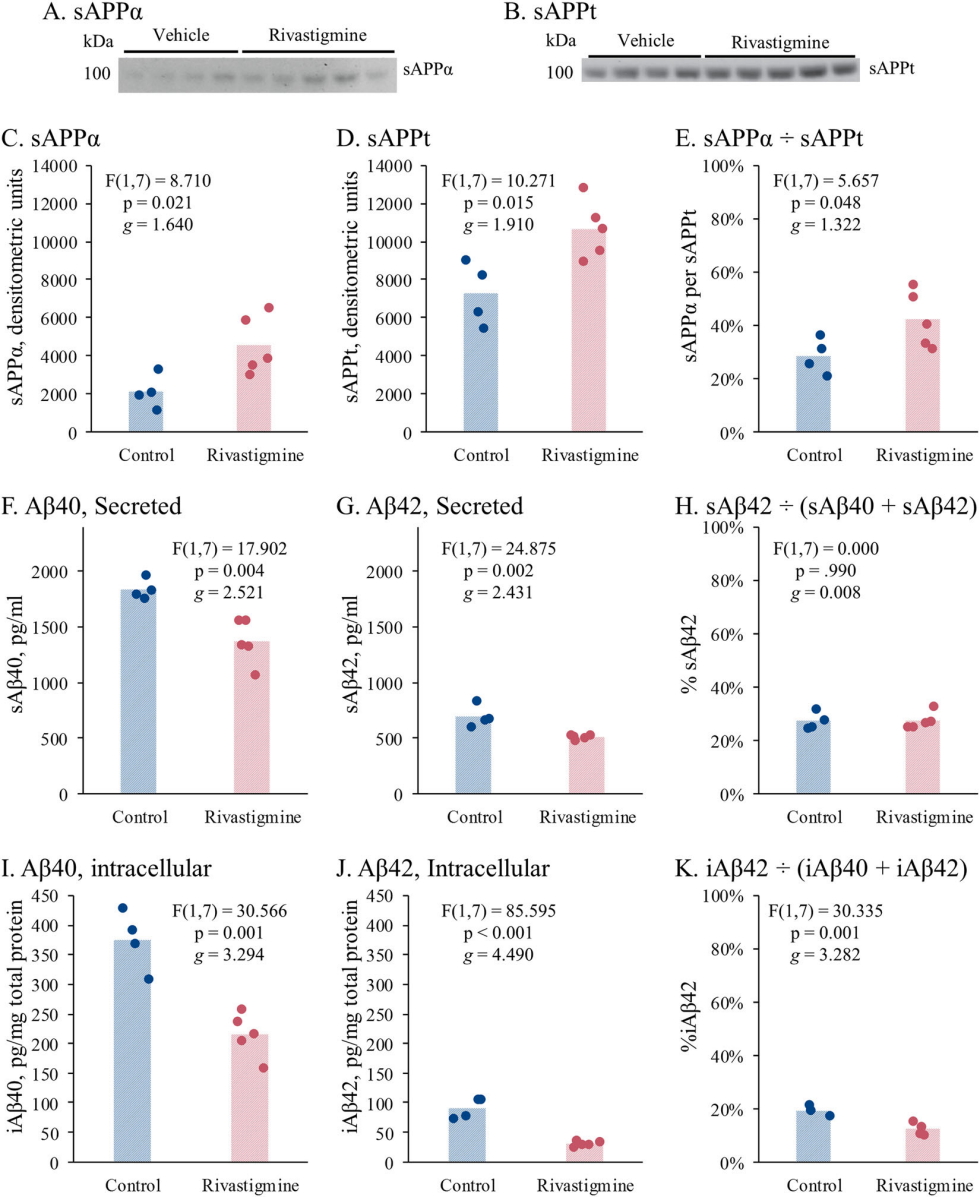
Effects of Rivastigmine on sAPPt, sAPPα, and Aβ levels in hippocampus of 3xTg mice. Alzheimer’s 3xTg mice were treated with rivastigmine for 21 days and hippocampi dissected and snap frozen. Secreted fractions from the hippocampi samples were extracted using detergent-free buffer or buffer with detergent. Reported statistics are from *t* test. Detergent-free extracts were assayed with western immunoblot and ELISA while detergent-containing extracts were assayed with ELISA. **a** Western blot of sAPPα. **b** Western blot of sAPPt. **c** sAPPα levels. **d** sAPPt levels. **e** Ratio of sAPPα to sAPPt. **f** Secreted (detergent-free) Aβ40 (sAβ40). **g** sAβ42. **h** Ratio of sAβ42 to sAβ40 + sAβ42. **i** Intracellular Aβ40 (iAβ40). **j** iAβ42. **k** Ratio of iAβ42 to iAβ40 + iAβ42.

Western immunoblot analyses of sAPPα (Fig. 4a) and sAPPt (Fig. 4b) in M-PER buffer soluble intracellular extract from hippocampal lysates of 3×Tg mice revealed that both (Fig. 4c, d) were elevated upon rivastigmine treatment vs. untreated mice. Likewise, the ratio of sAPPα to sAPPt was elevated after Rivastigmine treatment (Fig. 4e).

### Rivastigmine treatment decreased levels of intracellular and secreted Aβ peptides in the hippocampus of 3xTg mice

Quantification of intracellular and secreted Aβ40 and Aβ42 levels by ELISA revealed a decline in the hippocampus of rivastigmine-treated 3×Tg mice (Fig. 4f–g, i–j). Rivastigmine did not affect the sAβ42 to total sAβ ratio (Fig. 4h), but reduced the ratio of intracellular Aβ42 (iAβ42) to total iAβ (Fig. 4k).

### Rivastigmine increased levels of mature ADAM-9, ADAM-10, and pro-ADAM-10 in PHB cultures. TAPI increased levels of ADAM-9 and pro-ADAM-10, but decreased levels of mature ADAM-10

**Fig. 5:**
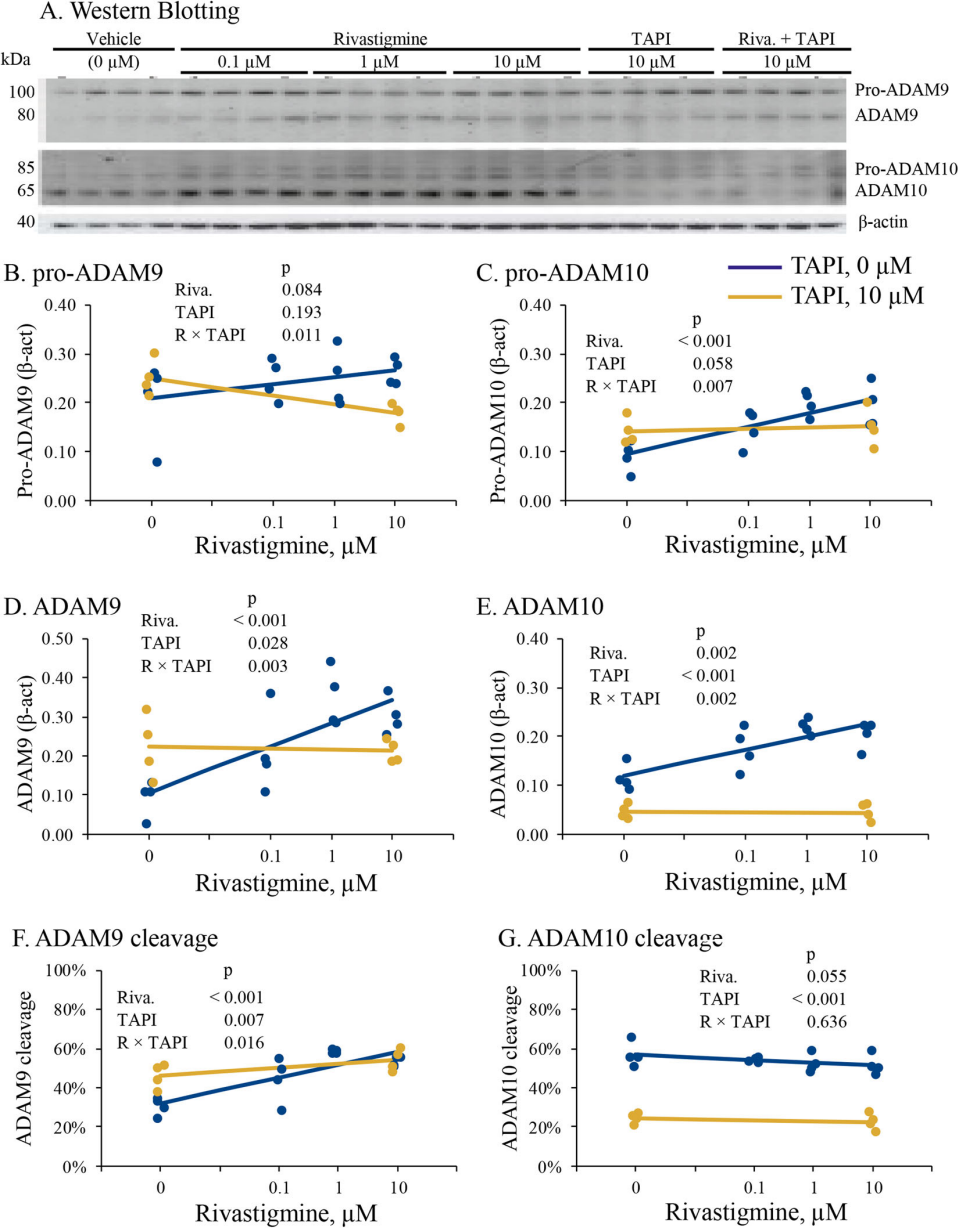
Changes induced by rivastigmine and TAPI in levels of different pro- and mature ADAM proteins and ADAM processing in human cultures, interaction effects. Primary human brain (PHB) cultures were co-treated with rivastigmine and TAPI as described in the text. Figure shows effects of rivastigmine and TAPI. Western immunoblotting was carried out on cell lysates to evaluate alterations of pro- and mature ADAM proteins by rivastigmine treatment and TAPI treatment. Reported statistics are vs. log(dose + 1). **a** Western blotting showing different proteins. **b** pro-ADAM-9. **c** pro-ADAM-10. **d** Mature ADAM-9. **e** Mature ADAM-10. **f** ADAM-9 processing, measured as mature ÷ (mature + pro). **g** ADAM-10 processing.

Western immunoblotting of CL samples from primary PHB cultures (Fig. 5a) revealed significant increases in levels of intracellular mature ADAM-9 (Fig. 5d) and ADAM-10 (Fig. 5e) in rivastigmine-treated samples vs controls as well as significant elevation in pro-ADAM-10 (Fig. 5b) levels. There was no evidence of change in pro-ADAM-9 (Fig. 5b). TAPI treatment, on the other hand, increased mature ADAM-9 (Fig. 5d) and pro-ADAM-10 (Fig. 5e), but resulted in significant reduction of mature ADAM-10 (Fig. 5e). Rivastigmine enhanced apparent cleavage of ADAM-9 (Fig. 5f), while TAPI enhanced cleavage of ADAM-9 (Fig. 5f) but significantly diminished maturation of ADAM-10 (Fig. 5g). ADAM-17 densitometry was saturated and unable to be reliably quantified (Fig. 1). Two-way modeling of the data revealed that several rivastigmine effects were altered by addition of TAPI. Notably, TAPI significantly interfered with rivastigmine vs. precursor and mature ADAM-9 and 10 (Fig. 5a–e). TAPI reversed rivastigmine effects on maturation of ADAM-9 (Fig. 5e), but not ADAM-10 maturation (Fig. 5f). ANOVA outcomes summarized in Supplementary Table S3.

### Rivastigmine treatment associated with increased levels of sAPPα in postmortem brain samples

**Fig. 6:**
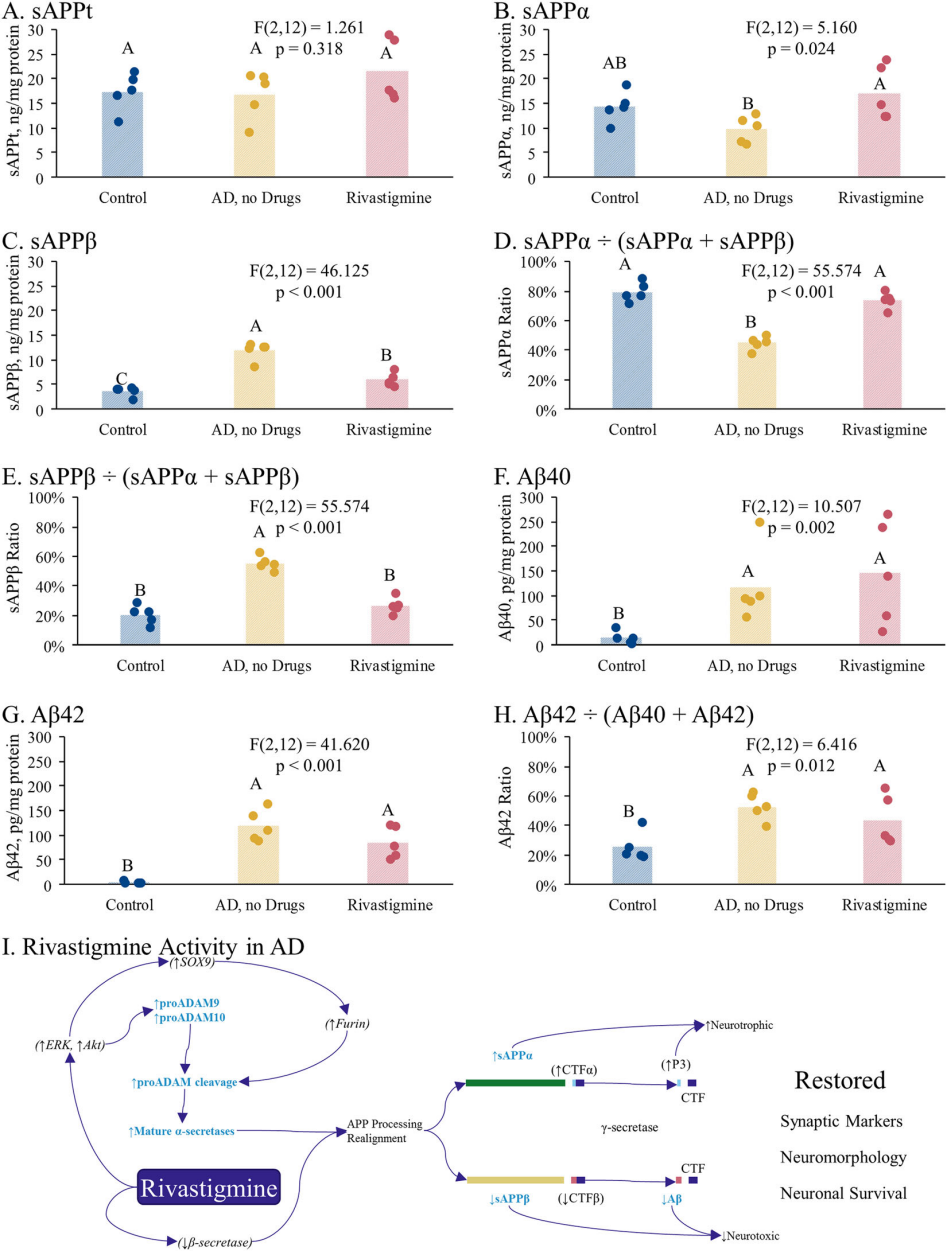
Effects of rivastigmine in AD brain samples. Postmortem brain tissue (Brodmann Areas 21/22) extracts obtained from non-AD controls, AD patients who were treated with rivastigmine, and AD patients who were given no drug treatments for AD were assayed by separate ELISAs for levels of sAPPα, sAPPt, Aβ40, and Aβ42 in the secreted fraction. ANOVAs were followed by simultaneous pairwise comparisons. Reported p-values in figure are for omnibus ANOVA. Pairwise comparisons were performed at α = 0.05. Groups sharing letters do not significantly differ from each other at this level of significance. Effect sizes of pairwise differences are expressed as Hedge’s *g*. **a** sAPPt. **b** sAPPα. **c** sAPPβ. **d** sAPPα ÷ (sAPPα + sAPPβ). **e** sAPPβ ÷ (sAPPα + sAPPβ). **f** Secreted Aβ40. **g** Secreted Aβ42. **h** Aβ42 ÷ (Aβ40 + Aβ42). **i** Rivastigmine restoration of neuronal and synaptic viability and function through realigning APP processing pathways. Bold, blue text indicates protein or peptide differences specifically reported in this paper. *Italicized* text indicates cited pathways/factors. Rivastigmine has the effect of reducing BACE1 (β-secretase) levels^92^. In addition, it stimulates ERK and Akt activation^108^. ERK, in particular, regulates the ADAM-9 and 10 α-secretase proteins^93–95,109^ and the SOX9 transcription factor^98,99^. SOX9 regulates the proprotein convertase Furin^97^, which converts proADAM-9 and 10 into mature (active) forms^96^. Thus, rivastigmine activity results in enhancement of levels of mature (active) α-secretase proteins. This enhancement combined with depression of β-secretase drives APP processing toward the “non-amyloidogenic” neuroprotective/neurotrophic pathway. Increase in neurotrophic APP cleavage products results in restoration or enhancement of synaptic markers (such as synaptophysin), neuromorphology, and overall neuronal survival.

Analyses of postmortem brain tissue extracts revealed no changes in sAPPt (Fig. 6a) but a significant rise in sAPPα (Fig. 6b) in samples from patients treated with rivastigmine vs. those who had not received any AD medication. sAPPβ levels dropped significantly with rivastigmine treatment (Fig. 6c). Ratios of sAPPα or sAPPβ to sAPPt followed similar trends (Fig. 6d, e). Brain levels of Aβ (1–40 and 42) were elevated overall in AD subjects compared to non-AD controls (Fig. 6f, g). However, there was no significant difference in Aβ levels when we compared those treated with rivastigmine vs. nontreated AD patients (Fig. 6g, h). Effect sizes (Hedge’s *g*) for pairwise comparisons are in Supplementary Table S4.

### ADAM-10 may be a particularly influential α-secretase in PHB

Correlation of biomarker levels (Supplementary Table S5), revealed that both mature ADAM-9 and ADAM-10 levels significantly (*p* ≤ 0.05, adjusted for multiple comparisons by false discovery rate^42^) correlated with any APP metabolite levels. ADAM-9 levels only significantly correlated (r = −0.584) with Aβ40, while ADAM-10 levels correlated significantly with sAPPt (r = 0.685), sAPPα (r = 0.834 or r = 0.849, Western and ELISA), sAPPβ (r = −0.747), CTFβ (r = −0.691). In addition, whereas levels of pro- and mature ADAM-9 correlated with each other (r = 0.595), correlation between pro- and mature ADAM-10 levels (r = 0.555) was not significant.

## Discussion

National Institutes of Health, USA (NIH) started an initiative to test (https://grants.nih.gov/grants/guide/pa-files/PAR-18-909.html) approved drugs with known safety profiles for alternate treatments due to the multiple clinical benefits of most chemical entities. It is, therefore, important to undertake basic mechanistic studies of drugs already approved for clinical use. Most drugs are studied in different cellular and animal models, mostly transgenic mice, and further mechanisticy study effectively stops if the data yield desired results. Human AD trials also focus on specific cognitive or institutionalization goals, and upon achieving the desired outcomes, the focus shifts to marketing and distributing the drug. There is generally little post-approval followup to determine the effects of the drugs on AD pathology, which forms the basis of its progression. Given that at least Exelon is administered to nearly every AD patient in the USA, it is critical to understand long-term effects of the agent on patients' brains and effects of cholinergic overload. This study directly evaluates the brains of rivastigmine-treated AD patients and AD patients not treated with any ChEI and compares both to cognitively normal subjects.

In addition, anti-AD drugs are extensively studied in the context of their “targets”, be they cholinergic systems, NMDA, microtubule-associated protein tau, or Aβ. Additional work may be done on effects on tau or Aβ, but additional processing enzymes and metabolites are generally not also included in any reports, even if those effects may play a role in disease progression. For example, the ChEI (−)-Phenserine has APP and Aβ lowering properties in cell culture, animal models^20^, and AD subjects^21^. Donepezil elevates sAPPα and reduces Aβ in culture by upregulating sorting nexin protein 33 (SNX33)^22^. Our study is a comprehensive look at rivastigmine effects on sAPPα and Aβ plus the α-secretases, ranging from cell culture, rat brain, human primary brain cultures, and adult human brains. Regarding such comprehensive study, no other study has reported the effects of an anti-AD drug at distant points along the lifespan, specifically fetal brain-origin primary cultures and elderly subject brains.

This study has revealed a potentially novel disease modifying activity for AD and did so through comprehensive analysis of the Aβ biogenesis pathway and its yield. The cholinesterase inhibiting effects of rivastigmine are valuable, as it targets butyrylcholinesterase, which is increased in AD, but they are short-term and temporary. Our study suggests that rivastigmine’s “beneficial side effects” may indicate its utility in treating AD at earlier, prodromal stages, such as MCI.

We demonstrated that rivastigmine promotes α-secretase processing by upregulating levels of ADAM-9, -10, and -17, and the precursors of ADAM-10 and -17, as well as sAPPα. This increase in processing by the non-amyloidogenic pathway accompanies a decline in levels of sAPPβ, Aβ40, and Aβ42. Rivastigmine elevated sAPPα and reduced potentially neurotoxic sAPPβ and Aβ at concentrations as low as 100 nM, which, unlike prior studies, has clinical relevance^43^. Notably, rivastigmine-induced activity was reversed by the addition of a nonselective ADAM inhibitor, TAPI. Further, TAPI without rivastigmine resulted in significantly elevated levels of mature ADAM-9. However, it significantly reduced levels of mature ADAM-10. Human postmortem brain sAPPα levels were also elevated in AD patients treated with rivastigmine, compared to untreated AD patients, which confirms elevation in sAPPα levels observed in PRBC following rivastigmine treatment^17^. We have characterized a novel, potentially disease-modifying, function for rivastigmine that may be independent of its anticholinesterase activities. Should this function exist in non-cholinergic rivastigmine derivatives it may provide a fruitful avenue of early pharmaceutical intervention without the unwanted dose- limiting effects of cholinesterase modification.

On its face, this appears to be untenable, given that the comprehensive “Investigation Into Delay to Diagnosis of Alzheimer’s Disease With Exelon” (InDDEx) study reported no significant benefit of rivastigmine to delay progression from MCI to AD^44^. However, stratification of the same study’s outcomes by gender revealed a significant delay in progression for women treated with rivastigmine^45^. Furthermore, non-blind trial patient studies have shown greater efficacy of early administration of rivastigmine for women than for men^46,47^. In particular, one such study examined anatomical changes in patient brains and explicitly suggested that rivastigmine “treatment also significantly reduced ventricular expansion, whole-brain atrophy rate and white matter loss in female BuChE wildtype animals, suggesting a possible disease-modifying effect.” based on brain pathology difference^46^. Mechanistic studies in animals showed that males eliminated rivastigmine 2.5 times more quickly than did females^48^. Estrogen modifies the processing of APP^49^. An estrogen receptor 1 (ESR1) haplotype modified the response to rivastigmine in a human study^47^. Animal studies suggest that testosterone may also play a role. Testosterone may interfere with rivastigmine entry into the brain^50^, and greater clearance of rivastigmine in male animals was attributed to testosterone^48^. Amelioration of induced memory deficits by rivastigmine differed by sex, but this was altered by orchidectomy and not ovariectomy^51^.

ChEIs are widely used to treat the symptoms of AD^52,53^, with four agents (tacrine, donepezil, rivastigmine, and galantamine) approved by the U.S. FDA and the European Medicines Agency (EMA). Each of these ChEIs varies from one another in how they inhibit ChE, and in their pharmacokinetics, safety, and long-term efficacy^54^. Furthermore, selective butyrylcholinesterase inhibition increased brain acetylcholine, augmented learning, and reduced Aβ peptide in rodents. This suggests that reversible inhibition of brain BChE could represent a treatment of AD, improving cognition and modulating neuropathological markers of AD.

A hypothesis for AD pathogenesis posits a shift in the processing of APP towards the amyloidogenic route or a decline in Aβ brain clearance that causes excessive accumulation of Aβ, and/or a shift in the ratio of Aβ species to favor the Aβ42 form^55^, although further truncated forms of Aβ may also play a role^56,57^. The reported upregulation of BACE1 during aging and AD may partly underlie this brain change^58–60^. There is support for a decline in α-secretase activity during AD. Reductions in CSF sAPPα levels exist in sporadic as well as nonsporadic AD^61,62^, and positive correlations exist between declines sAPPα levels and reduced cognitive performance in AD subjects^62^ and normal aged rats^63^. However, other studies have reported unchanged levels of sAPPα in CSF during sporadic AD^64–67^, with decreases only evident in advanced AD^67^; suggesting a need for further evaluation concerning disease staging^68^. sAPPα possesses neurotrophic and neuroprotective actions across cellular and in vivo models^68^. Thus, augmenting brain sAPPα levels has become a therapeutic strategy across neurodegenerative disorders^61,69^

Our study builds on previous findings suggesting that ChEIs can possess APP-modulating properties^70^. As an example, the acetyl-ChEI phenserine dose-dependently reduces the synthesis of total APP post-transcriptionally, via an iron regulatory element in the 5′−untranslated region (UTR) of APP mRNA^19,20^. In contrast, other ChEIs such as (−)−physostigmine lack action on APP^71^.

To evaluate the translational relevance of these cell culture findings in an in vivo system, we treated 3×Tg AD model mice with rivastigmine with a clinically relevant dose equivalent to approximately 5 mg rivastigmine for an 80 kg human, following normalization of body surface area between species, in line with FDA guidelines^72^. A significant rise in brain levels of sAPPα resulted, accompanied by a decrease in Aβ40 and 42.

We quantified sAPPα levels in postmortem human brain samples obtained from AD subjects who had been administered rivastigmine and compared these to samples from patients who did not receive any ChEI drug. Notably, we observed higher levels of sAPPα in the secreted fraction of brain samples from subjects exposed to rivastigminewithout a reduction in Aβ. Such failure to see changes in Aβ levels in late-stage AD upon rivastigmine treatment is readily explained by the large Aβ accumulation over years or decades, which may mask any changes in Aβ production. A complete shut off of all Aβ production would be unlikely to quickly produce an accompanying reduction in measured Aβ levels in the brain, even if Aβ clearance mechanisms functioned at normal levels, and in AD, Aβ clearance is impaired^73^.

sAPPα is neurotrophic^74–82^. The intraventricular administration of sAPPα enhances memory function of mice^83^, and sAPPα mediates numerous APP-mediated actions on brain development and cognition^68,74,84–86^. A mutation within the α-secretase cleavage site of human APP (APP770K687N) leads to reduced sAPPα production and elevation of Aβ and causes early onset dementia^87^. Activity-attenuating mutations within the prodomain of human ADAM-10 gene link to AD^88,89^. It is noteworthy, therefore, that in AD mouse models, rivastigmine treatment reduced Aβ-related brain pathology^90^ and cognitive dysfunction, specifically related to Aβ load^91^.

Rivastigmine nanoformulations significantly inhibit BACE1 mRNA levels, which may, in turn, increase APP processing via the α-secretase pathway^92^. We show, herein, that rivastigmine treatment increases levels of ADAM-9 and ADAM-10. Among potential stimulatory pathways induced by rivastigmine, ADAM-10 transcription is elevated by altering extracellular signal-related kinase (ERK) 1/2 phosphorylation^93,94^. Inhibition of ERK reduces activity of ADAM-9^95^. The ADAM proproteins are processed by the proprotein convertase furin^96^. Furin is regulated by transcription factor SOX9^97^. SOX9 is regulated by the MAPK/ERK pathway^98^, including the participation of ERK^99^. We propose, therefore, a testable and mechanistic pathway for the non-ChEI-mediated neuroprotective and neurotrophic activity of rivastigmine (Fig. 6i). Several elements of this model are not explicitly tested in the current study. We are proposing a hypothesis based on our work along with others’ in the field.

Another activity of APP is its contribution to Fe (iron) homeostasis in neuronal cells^100–103^. In particular, this operates through a site in the APP 5′-UTR that binds iron response protein 1 (IRP1)^100,101,103^, interleukin 1 (IL1)^102,104,105^, and microRNA-346^100^. This “FeAR (Fe, APP, RNA) nexus” may also play a feedback role in α-secretase processing of APP, given that Fe also modulates α-secretase cleavage of APP^106^, specifically to increase sAPPα cellular retention and inhibit BACE1 activity^107^.

Rivastigmine alters activity of α- and β-secretases. It reduces β-secretase through an unknown mechanism^92^. However, rivastigmine nanoformulation can produce significant inhibition in mRNA levels of BACE1^92^. Rivastigmine stimulates the activation of both ERK and Akt^108^. ERK regulates ADAM-9, -10, and -17 at multiple levels^93–95,109^ and regulates transcription factor SOX9^98,99^. SOX9 regulates Furin^97^, which processes the ADAM-9, -10, and -17 proproteins^96^, although we only found rivastigmine stimulation of proADAM-9 cleavage. Ultimately, levels of the mature α-secretases increase, corresponding to increased α-secretase activity. The combination of reduced β- and enhanced α-secretase drives APP processing toward neurotrophic/protective sAPPα and related products. These could stimulate restoration of synaptic markers, such as synaptophysin, and maintain and promote healthy neuromorphology and neurosurvival^17,18,68,80,83^.

Rivastigmine is a ChEI. Its administration is expected to result in net increases in acetylcholine (and butyrylcholine)^14,110^. Acetylcholine stimulates nicotinic and muscarinic receptors, and such stimulation further stimulates α-secretase activity and reduces Aβ production^111^. Thus, an M1-receptor pathway may explain our findings. In particular, the M1 receptor agonists AF102B or dicyclomine resulted in activation of PKCα and ERK, resulting in increased levels of ADAM-17 and a shift in APP processing toward the non-amyloidogenic pathway^112^. However, treatment of primary cortical neurons with the M1 agonist carbachol did not alter levels of ADAM-10, ADAM-17, or BACE1 in their brains^113^, and M1 receptor knockout likewise did not alter ADAM-10 or ADAM-17 levels^113^. The neuroprotective extract of *Withania somnifera* root reversed effects of a scopolamine memory loss model^114^ and operated through the M1 receptor^115^. While we propose an M1-independent model, we understand that a cholinergic alternative may exist.

Thus far, all Aβ-based clinical trials for AD treatment have failed. Would this mean rivastigmine’s potential disease-modifying activity is foredoomed as a treatment? We would say it is still viable because (1) those Aβ-related treatments that have failed in clinical trial were administered after amyloid plaque accumulation, when plaque may have reached a point wherein clearance mechanisms were simply no longer up to the task. In addition, AD brains accumulate Aβ, tau, and a number of other proteins, suggesting the failure of shared protein turnover pathways^4,116^. However, pathogenesis of AD is now recognized as beginning well before obvious “pathology” can be detected^117–119^. In addition, the potential disease-modifying activity of rivastigmine may be due not so much to reduction of Aβ but stimulation of neurotrophic sAPPα production and other effects of ADAM proteins. Any accompanying Aβ reduction may be a fortuitous happenstance. On the other hand, rivastigmine’s potential disease-modifying activity could be due both to cholinergic and non-cholinergic pathway modifications, making it a “one-drug cocktail”.

In conclusion, we have evaluated the impact of rivastigmine on APP processing across neuronal cell lines and primary mixed cell human brain cultures, within the hippocampus of a well-characterized AD animal mouse model (3×Tg mice), and in human postmortem brain tissue. The mouse dosing we used was several times that currently used for humans (4–5 times transdermal dose, 9–11 times oral dose, mg/kg basis). Nevertheless, it is a commonly-used dose for animal studies^91,120^. Efficacy and side effects must be titrated for each individual species. We consistently observed evidence that rivastigmine treatment associated with α-secretase activity: Elevated levels of the active forms of ADAM-9, -10, and -17, as well as sAPPα. If appropriately optimized and harnessed, this drug activity could impact Alzheimer’s disease progression via a drug whose tolerability and efficacy is already established for the symptomatic treatment of AD.

## Supplementary information

Supplemental Material

## Data Availability

The datasets generated during the current study are available from the corresponding author on reasonable request.
