# Correction: Rivastigmine modifies the α-secretase pathway and potentially early Alzheimer’s disease

**DOI:** 10.1038/s41398-020-0757-2

**Published:** 2020-03-02

**Authors:** Balmiki Ray, Bryan Maloney, Kumar Sambamurti, Hanuma K. Karnati, Peter T. Nelson, Nigel H. Greig, Debomoy K. Lahiri

**Affiliations:** 1 Department of Psychiatry, Laboratory of Molecular Neurogenetics, Indiana University School of Medicine, Indianapolis, IN 46202 USA; 2 Indiana Alzheimer Disease Center, Indiana University School of Medicine, Indianapolis, IN 46202 USA; 3 Department of Neurosciences, Medical University of South Carolina, Charleston, 29425 SC USA; 4 National Institute on Aging, Drug Design and Development Section, Bethesda, MD 20892 USA; 5 Sanders-Brown Center on Aging, University of Kentucky, Lexington, KY 40536 USA; 6 Department of Medical and Molecular Genetics, Indiana University School of Medicine, Indianapolis, IN 46202 USA

**Keywords:** Health sciences, Diseases, Psychiatric disorders

**Correction to: Translational Psychiatry**

10.1038/s41398-020-0709-x published online 03 February 2020

The original article contained few errors: Hanuma K. Karnati’s name was misstated in the author list, references 2 and 55 referred to the wrong sources, and the authors wanted to expand on their discussion of ChEIs on page 13. These errors have all been updated in the XML, PDF, and HTML versions of this article.

