# Supplementary material for: Rivastigmine modifies the α-secretase pathway and potentially early Alzheimer’s disease
Source: Transl Psychiatry. 2020 Feb 3;10:47. doi: 10.1038/s41398-020-0709-x (PMC7026402; doi:10.1038/s41398-020-0709-x)
Supplement: Supplementary file 1 — Supplemental Material [file 41398_2020_709_MOESM1_ESM.pdf]

Dose response analyses were accompanied by calculation of ω^2, which expresses proportion of variability accounted for in a less-biased way than does η^2 [1]. Fold-change per µM (FC) effect size was calculated as $\frac{x_0 - x_{max}}{x_0} \div D_{max}$, where x_0 = outcome at dose = 0µM, x_{max} = outcome at dose = 10µM, and D_{max} = maximum dose in µM, for mean values at minimum and maximum drug doses for single-treatment studies. Combined rivastigmine×TAPI tests used a "ΔFC" effect size, where FCs were calculated separately for each TAPI dose, and the FC for TAPI = 0µM was subtracted from the FC for TAPI = 10µM. Pairwise comparisons of human brain sample assays were accompanied with Hedge's *g* effect size [2].

1 Olejnik, S. & Algina, J. Generalized eta and omega squared statistics: measures of effect size for some common research designs. *Psychol. Methods* **8**, 434-447, doi:10.1037/1082-989X.8.4.434 (2003).

2 Hedges, L. V. & Olkin, I. *Statistical Methods for Meta-Analysis*. (Academic Press, 1985).

Table S1. ANOVA and Effect Sizes of Differentiated PC12 Cultures

Single Treatment Assays

Outcome	Treatment	F	p	ω^2	FC
CTG	Rivastigmine	101.843 (1,22)	<0.001	0.808	0.038
CTG	TAPI	15.666 (1,26)	<0.001	0.344	−0.009
sAPPα	Rivastigmine	101.607 (1,14)	<0.001	0.863	0.211
sAPPβ	Rivastigmine	19.347 (1,13)	<0.001	0.550	−0.034
Aβ40	Rivastigmine	11.474 (1,14)	0.004	0.396	−0.014

Rivastigmine/TAPI co-Treatment

CTG	Effect	F	p	ω^2	FC/ΔFC
	Rivastigmine	55.910 (1,20)	<0.001	0.530	0.008
	TAPI	0.002 (1,20)	0.969	0.000	0.000
	Riva × TAPI	26.708 (1,20)	<0.001	0.248	−0.010

sAPPα	Effect	F	p	ω2	FC/ΔFC
	Rivastigmine	14.644 (1,68)	<0.001	0.129	+0.048
	TAPI	8.769 (1,68)	0.004	0.074	−0.034
	Riva × TAPI	12.966 (1,68)	<0.001	0.114	−0.036

Table S2. ANOVA and Effect Sizes of PHB Culture APP metabolites

hAPP	Effect	F	p	ω2	FC/ΔFC
	Rivastigmine	0.626, (1, 20)	0.438	0.000	0.028
	TAPI	0.764, (1, 20)	0.393	0.000	−0.024
	Riva × TAPI	1.191, (1, 20)	0.288	0.008	−0.051
sAPPt	Effect	F	p	ω2	FC/ΔFC
	Rivastigmine	0.208, (1, 20)	0.654	0.000	0.006
	TAPI	1.417, (1, 20)	0.248	0.014	−0.015
	Riva × TAPI	6.466, (1, 20)	0.019	0.188	−0.049
sAPPα (WB)	Effect	F	p	ω2	FC/ΔFC
	Rivastigmine	11.198, (1, 20)	0.003	0.145	0.138
	TAPI	25.127, (1, 20)	<0.001	0.344	−0.074
	Riva × TAPI	12.802, (1, 20)	0.002	0.168	−0.175
sAPPα (ELISA)	Effect	F	p	ω2	FC/ΔFC
	Rivastigmine	76.483, (1, 20)	<0.001	0.421	0.069
	TAPI	77.262, (1, 20)	<0.001	0.426	−0.039
	Riva × TAPI	4.474, (1, 20)	0.047	0.019	−0.070
sAPPβ	Effect	F	p	ω2	FC/ΔFC
	Rivastigmine	33.582, (1, 20)	<0.001	0.469	−0.033
	TAPI	0.262, (1, 20)	0.614	0.000	0.004
	Riva × TAPI	14.675, (1, 20)	0.001	0.197	0.024
CTFβ	Effect	F	p	ω2	FC/ΔFC
	Rivastigmine	7.728, (1, 20)	0.012	0.209	−0.031
	TAPI	0.993, (1, 20)	0.331	0.000	0.015
	Riva × TAPI	2.504, (1, 20)	0.129	0.047	0.014
Aβ40	Effect	F	p	ω2	FC/ΔFC
	Rivastigmine	24.015, (1, 20)	<0.001	0.406	−0.048
	TAPI	0.439, (1, 20)	0.515	0.000	−0.007
	Riva × TAPI	11.165, (1, 20)	0.003	0.180	0.048
Aβ42	Effect	F	p	ω2	FC/ΔFC
	Rivastigmine	19.896, (1, 20)	0.000	0.337	−0.038
	TAPI	2.999, (1, 20)	0.099	0.036	−0.017
	Riva × TAPI	12.107, (1, 20)	0.002	0.198	0.045
CTG	Effect	F	p	ω2	FC/ΔFC
	Rivastigmine	0.400, (1, 20)	0.534	0.000	−0.004
	TAPI	7.955, (1, 20)	0.011	0.231	−0.018
	Riva × TAPI	0.777, (1, 20)	0.389	0.000	0.009

Table S3. ANOVA and Effect Sizes of PHB Cultures ADAM-9 and ADAM-10

pro-ADAM-9	Effect	F	p	ω^2	FC/ΔFC
	Rivastigmine	3.312, (1, 20)	0.084	0.068	0.028
	TAPI	1.818, (1, 20)	0.193	0.024	0.021
	Riva × TAPI	7.925, (1, 20)	0.011	0.203	−0.057
ADAM-9	Effect	F	p	ω^2	FC/ΔFC
	Rivastigmine	22.594, (1, 20)	0.000	0.354	0.232
	TAPI	5.577, (1, 20)	0.028	0.075	0.115
	Riva × TAPI	11.789, (1, 20)	0.003	0.177	−0.237
A9 Maturation	Effect	F	p	ω^2	FC/ΔFC
	Rivastigmine	31.077, (1, 20)	<0.001	0.442	0.083
	TAPI	8.994, (1, 20)	0.007	0.117	0.045
	Riva × TAPI	6.993, (1, 20)	0.016	0.088	−0.065
pro-ADAM-10	Effect	F	p	ω^2	FC/ΔFC
	Rivastigmine	22.117, (1, 20)	0.000	0.376	0.120
	TAPI	4.047, (1, 20)	0.058	0.054	0.051
	Riva × TAPI	8.949, (1, 20)	0.007	0.142	−0.114
ADAM-10	Effect	F	p	ω^2	FC/ΔFC
	Rivastigmine	4.166, (1, 20)	0.055	0.019	0.091
	TAPI	141.542, (1, 20)	<0.001	0.842	−0.061
	Riva × TAPI	0.231, (1, 20)	0.636	0.000	−0.093
A10 Maturation	Effect	F	p	ω^2	FC/ΔFC
	Rivastigmine	12.909, (1, 20)	0.002	0.163	−0.010
	TAPI	26.698, (1, 20)	<0.001	0.352	−0.057
	Riva × TAPI	12.425, (1, 20)	0.002	0.156	0.002

Table S4. Pairwise effect sizes (Hedge's *g*), human brain samples

Comparison	sAPPt	sAPPα	sAPPβ	sAPPα ratio[a]	sAPPβ ratio[b]	Aβ40	Aβ42	Aβ42 ratio[c]
Control vs. AD, no drugs	0.12	1.41	5.23	5.49	5.49	1.75	4.60	2.69
Control vs. Rivastigmine	0.71	0.55	1.82	0.92	0.92	1.59	3.26	1.20
Rivastigmine vs. AD, no drugs	0.75	1.51	3.37	5.17	5.17	0.28	0.95	0.64

[a]sAPPα ÷ (sAPPα + sAPPβ)
[b]sAPPα ÷ (sAPPα + sAPPβ)
[c]Aβ42 ÷ (Aβ40 + Aβ42)

Table S5. Correlation of β-actin normalized signals, Rivastigmine × TAPI

		iAPPt[ab]	sAPPt[b]	sAPPα[b]	sAPPα[c]	sAPPβ[c]	CTFβ[c]	Aβ40[c]	Aβ42[c]	ADAM-9		ADAM-10		A17[d]
										pro[b]	mature[b]	pro[b]	matur[b]e	pro[b]
**sAPPt[b]**		**0.630**												
**sAPPα[b]**		**0.466**	**0.509**											
**sAPPα[c]**		**0.451**	0.510	**0.875**										
**sAPPβ[c]**		−0.242	−0.362	**−0.737**	**−0.687**									
**CTFβ[c]**		−0.184	−0.238	−0.506	−0.567	**0.584**								
**Aβ40[c]**		−0.334	−0.488	**−0.637**	**−0.665**	**0.777**	0.445							
**Aβ42[c]**		−0.134	−0.223	−0.471	−0.496	**0.608**	0.350	**0.761**						
**ADAM-9**	**pro[b]**	0.327	**0.583**	0.363	0.329	−0.490	−0.251	**−0.643**	−0.377					
	**mature[b]**	0.437	0.305	0.399	0.400	−0.523	−0.484	**−0.584**	−0.504	**0.595**				
**ADAM-10**	**pro[b]**	0.220	0.237	0.423	0.332	**−0.649**	**−0.622**	−0.579	−0.533	0.424	**0.728**			
	**mature[b]**	0.495	**0.685**	**0.834**	**0.849**	**−0.747**	**−0.670**	**−0.691**	−0.452	0.444	0.510	0.550		
**ADAM-17**	**pro[b]**	0.284	0.367	0.277	0.252	−0.157	−0.153	−0.436	−0.469	**0.674**	**0.701**	0.326	0.294	
	**mature[b]**	0.169	0.400	0.443	0.435	−0.234	−0.285	−0.374	−0.537	0.380	0.481	0.344	0.421	**0.777**

[a]Boldface indicates significance at adjusted $p \leq 0.05$.

[b]Measured by semi-quantitative western blot.

[c]Measured by ELISA

S1

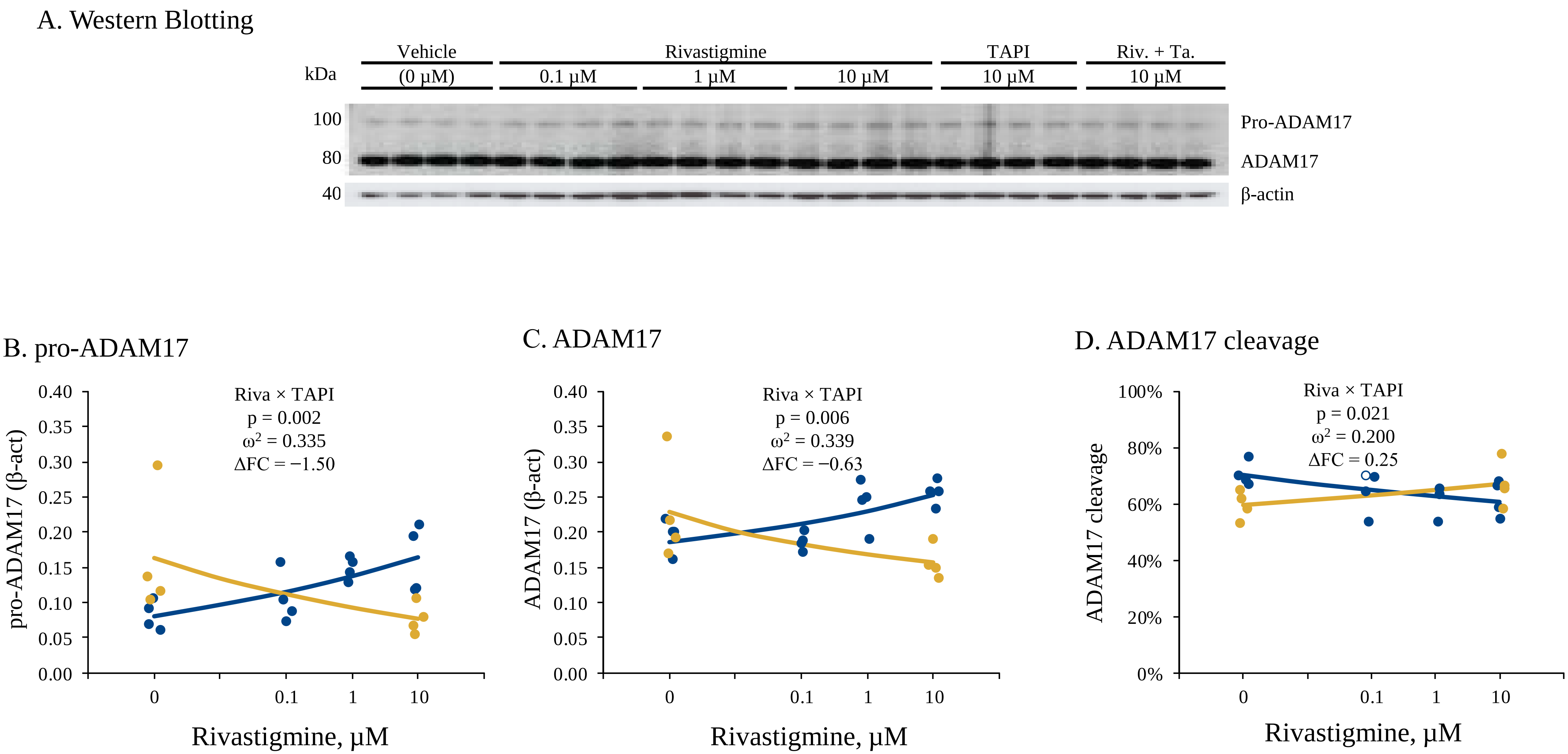
A. Western Blotting
Vehicle
(0 μM)
Rivastigmine
0.1 μM
1 μM
10 μM
TAPI
10 μM
Riv. + Ta.
10 μM
kDa
100
80
40
Pro-ADAM17
ADAM17
β-actin
B. pro-ADAM17
pro-ADAM17 (β-act)
Riva × TAPI
p = 0.002
ω2 = 0.335
ΔFC = −1.50
Rivastigmine, μM
C. ADAM17
ADAM17 (β-act)
Riva × TAPI
p = 0.006
ω2 = 0.339
ΔFC = −0.63
Rivastigmine, μM
D. ADAM17 cleavage
ADAM17 cleavage
Riva × TAPI
p = 0.021
ω2 = 0.200
ΔFC = 0.25
Rivastigmine, μM

Supplemental Figure S1. Changes induced by rivastigmine and TAPI in levels of different pro- and mature ADAM-17 and ADAM-17 processing in human cultures, interaction effects. Primary human brain (PHB) cultures were co-treated with rivastigmine and TAPI as described in the text. Figure shows effects of rivastigmine or TAPI alone. Western immunoblotting was carried out on cell lysates to evaluate alterations of pro- and mature ADAM-17 by rivastigmine treatment and TAPI treatment. Reported statistics are vs. log(dose + 1). A) Western blotting showing different proteins. B) pro-ADAM-17. C) mature ADAM-17. D) D) ADAM-17 processing, measured as mature ÷ (mature + proprotein).
